# An open-label prospective study of the real-life use of onabotulinumtoxinA for the treatment of chronic migraine: the REPOSE study

**DOI:** 10.1186/s10194-019-0976-1

**Published:** 2019-03-07

**Authors:** Fayyaz Ahmed, Charly Gaul, Juan Carlos García-Moncó, Katherine Sommer, Paolo Martelletti, on behalf of the REPOSE Principal Investigators

**Affiliations:** 1 Spire Hesslewood Clinic, Hull York Medical School, 28 Spindlewood, Elloughton, Brough, HU15 1LL UK; 2 Migraine and Headache Clinic Königstein, Königstein im Taunus, Germany; 3 Hospital de Galdacano, Vizcaya, Spain; 4 Allergan plc, Marlow, Buckinghamshire UK; 5 Department of Clinical and Molecular Medicine, Sapienza University of Rome, Rome, Italy; 6 Regional Referral Headache Centre, Sant’ Andrea Hospital, Rome, Italy

**Keywords:** OnabotulinumtoxinA, Chronic migraine, Effectiveness, Safety, Long-term, Clinical setting, Real world

## Abstract

**Background:**

The PREEMPT Studies established onabotulinumtoxinA as preventive treatment for adults with chronic migraine (CM). The purpose of the ***RE****al-life use of botulinum toxin for the symptomatic treatment of adults with chronic migraine, measuring healthcare resource utilisation, and* ***P****atient-reported* ***O****utcome****S*** *observed in practice* (REPOSE) Study was to observe real-life, long-term (24-month) use of onabotulinumtoxinA in adults with CM and report on the utilisation, effectiveness, safety, and tolerability.

**Methods:**

The REPOSE Study was a European, open-label, multicentre, prospective, noninterventional study. Patients received onabotulinumtoxinA approximately every 12 weeks according to their physician’s usual practice, guided by the summary of product characteristics (SPC). Patients were observed for 24 months after initiating onabotulinumtoxinA treatment. Outcome measures were collected at baseline and all administration visits and included onabotulinumtoxinA injection practices, headache-day frequency, Migraine-Specific Quality-of-Life Questionnaire (MSQ), EuroQol 5-Dimension Questionnaire (EQ-5D), and adverse drug reactions (ADRs) to evaluate safety/tolerability.

**Results:**

Of 641 patients enrolled, 633 received ≥1 dose of onabotulinumtoxinA for a total of 3499 treatment sessions. At baseline, mean (SD) age was 45.4 (11.7) years; patients were predominantly women (85.3%). Injection practices closely followed the SPC in mean dosage (155.1 U) and injection sites per session (31.4), with the exception of a prolongation of the recommended 12-week dosing interval, with 79.1% of patients receiving ≥1 treatment session that was > 13 weeks after the previous treatment session. Headache-day frequency was reduced from a baseline mean (SD) of 20.6 (5.4) to 7.4 (6.6) days at administration visit 8 (*P* < 0.001). Each MSQ domain (restrictive, preventive, and emotional) was significantly reduced from baseline through each administration visit *(P* < 0.001). The median EQ-5D total and health state scores were significantly improved from baseline through each administration visit (*P* < 0.001). Overall, 18.3% of patients reported an ADR; most were mild to moderate intensity, with only 1.3% of patients reporting a serious ADR. Eyelid ptosis (5.4%), neck pain (2.8%), and musculoskeletal stiffness (2.7%) were the most frequently reported.

**Conclusions:**

Long-term, real-world preventive treatment of CM with onabotulinumtoxinA showed effectiveness with a sustained reduction in headache-day frequency and significant improvement in quality-of-life measures. ADRs were mild to moderate, with no new safety concerns identified.

**Trial registration:**

Trial registration number: NCT01686581. Name of registry: ClinicalTrials.gov. URL of registry: https://clinicaltrials.gov/ct2/show/NCT01686581. Date of retrospective registration: September 18, 2012. Date of enrolment of first patient: July 23, 2012.

## Introduction

Chronic migraine (CM) and episodic migraine (EM) are part of the spectrum of migraine disorders. CM (generally defined as ≥15 headache days per month, with ≥8 days fulfilling migraine criteria) [1, 2] is a complex neurologic disorder with a global prevalence of approximately 1.4% to 2.2% [3]. It is associated with significant individual disability leading to societal and economic burden [4–7]. Studies have found that CM is associated with increased headache-related disability, psychiatric comorbidities, and greater financial and occupational burden compared with EM (defined as < 15 headache days per month) [4–9]. However, people with CM face barriers in receiving the proper medical management of their disease [8, 10]. Dodick et al. found that < 5% of people with CM were receiving appropriate care, which included consulting a healthcare professional, being accurately diagnosed, and being prescribed a treatment regimen [10]. Low consultation rates may be partially attributed to a lack of awareness of the general public to the available treatment options [11].

Currently, people with CM are generally treated with anticonvulsants (valproate, topiramate), antidepressants (amitriptyline), beta blockers (propranolol, metoprolol, timolol, bisoprolol), and angiotensin II receptor 1A blockers (candesartan), or onabotulinumtoxinA [12–14]. Most preventive treatment options are prescribed based on their effectiveness in EM but have limited or no evidence in CM and no CM-specific guidelines [12, 13]. Beta blockers and topiramate have been approved as migraine-preventive treatments but not specifically for CM [15–17]. Nonetheless, topiramate has been associated with acceptable efficacy in the prevention of headache in CM [18]. Despite the severity of the disease, only a minority of people with CM (40%) ever take preventive medication, and < 25% adhere to oral preventive medications 1 year after initiating treatment, primarily because of adverse events [1, 10].

OnabotulinumtoxinA (BOTOX®, Allergan plc, Dublin, Ireland) is approved in most European countries for reduction of headaches in adults with CM and as a preventive medication when patients are intolerant to or do not respond to other preventive medications [19]. Recommended treatment with onabotulinumtoxinA consists of intramuscular injections distributed among 7 head/neck muscle groups for a total dosage range of 155 to 195 U every 12 weeks [19].

The Phase III Research Evaluating Migraine Prophylaxis Therapy (PREEMPT) clinical program demonstrated the efficacy and safety of onabotulinumtoxinA over 56 weeks as a preventive treatment for adults with CM [20–22]. It was the largest placebo-controlled trial in this patient population and established the injection protocol and dosing specific to this product [23]. The Chronic Migraine OnabotulinuMtoxinA Prolonged Efficacy open Label (COMPEL) Study, an international, multisite, prospective, open-label study, supported the findings of the PREEMPT Study and provided evidence of the effectiveness and safety of longer-term use of onabotulinumtoxinA, extending to 108 weeks and using the PREEMPT injection paradigm without the “follow-the-pain” strategy [24]. Additionally, the findings from the COMPEL Study complemented the results of several single-site, longer-term studies conducted in routine clinical settings [25–30]. In one such prospective study in the United Kingdom, there were significant reductions in all outcome measures (headache and migraine days) and significant improvements in quality-of-life measures, such as the Headache Impact Test [30]. The ***RE****al-life use of botulinum toxin for the symptomatic treatment of adults with chronic migraine, measuring healthcare resource utilisation, and* ***P****atient-reported* ***O****utcome****S*** *observed in practice* (REPOSE) Study is a 24-month observational study that utilised patient- and physician-reported outcomes to assess the effectiveness and safety of real-life, long-term use of onabotulinumtoxinA for CM in multiple sites in Europe and evaluated the utilisation of onabotulinumtoxinA in routine clinical practice across Europe. In this report we present an overview of the real-world clinical utilisation of onabotulinumtoxinA and the associated effectiveness and safety in patients with CM.

## Methods

### Study design

The REPOSE Study is an open-label, prospective, noninterventional study to observe the real-life long-term use of onabotulinumtoxinA in adults diagnosed with CM. The study design and methodology have been reported previously [15]. In brief, patients were observed for a 24-month period following the start of onabotulinumtoxinA treatment; the planned total study duration was approximately 30 to 36 months [enrolment, baseline visit (including the first administration of onabotulinumtoxinA), and administration (admin) visits approximately every 3 months]. The study was initiated in July 2012 and completed in October 2016. Patients were enrolled from 78 centres in Germany, Italy, Norway, Russia, Sweden, Spain, and the United Kingdom.

Before study initiation, all investigators obtained ethical approval from their respective ethics committee. The study was conducted in accordance with the International Conference on Harmonisation Guideline for Good Clinical Practice. Informed consent was obtained from each patient before enrolment.

### Patient selection

Patients were eligible for inclusion if they were adults (≥18 years old) with a diagnosis of CM and were prescribed onabotulinumtoxinA by their physician for the prevention of headaches. Patients were excluded if they had received any botulinum toxin serotype within 26 weeks before enrolment, were concurrently enrolled in Botox CM Post-Authorization Safety Study (PASS), or were contraindicated for treatment with onabotulinumtoxinA. To optimally capture standard clinical practice, there were no other specific exclusion criteria; patients could have received acute or other preventive treatments before enrolment and could continue these treatments, changed or unchanged, throughout the study period. Investigators were to refer to the summary of product characteristics (SPC) for information on contraindications (section 4.3), warnings (section 4.4), and pregnancy and lactation (section 4.6) [19].

### Study treatment

#### OnabotulinumtoxinA utilisation

Treating physicians were trained according to the injection paradigm described in the SPC and the PREEMPT study protocol (ie, onabotulinumtoxinA 155 U spread over 31 injection sites at a dosing interval of 12 weeks, with discretion to administer an additional 40 U over 8 injection sites according to the follow-the-pain strategy to a maximum total dose of 195 U). However, physicians were not required to follow this paradigm [19]. At each visit, the total dose per treatment session and total number and location of injection sites were recorded for all patients.

#### Outcome measures

At administration visit 1 (the baseline visit), patient demographics, medical history, migraine history, previous/concomitant headache treatment, baseline values for outcome measures, and initiation of onabotulinumtoxinA treatment were collected. Outcome measures, including the use of acute and preventive headache medication, were collected at all administration visits. Administration visits were defined as visits in which onabotulinumtoxinA was injected. Outcome measures for administration visits through to administration visit 8 are reported herein, reflecting the expected number of treatment sessions administered during a 24-month period based on a 12-week administration interval. The frequency of headache days was determined using the patient-reported estimated number of days in a month with a headache lasting ≥4 h. Follow-up visits included any visit after the baseline visit and did not necessarily include the administration of onabotulinumtoxinA.

The Migraine-Specific Quality-of-Life Questionnaire (MSQ) v2.1 is a 14-item questionnaire measuring the impact of migraines on the respondent’s quality of life and daily activities [31, 32]. The scale consists of 3 domains: (1) role-function restrictive assesses limitations to the patient’s daily social and work-related activities; (2) role-function preventive assesses how migraines prevent these activities; and (3) emotional function assesses the patient’s emotions associated with migraines. Each item is evaluated on a 6-point scale using the following scores: 1 (none of the time), 2 (a little bit of the time), 3 (some of the time), 4 (a good bit of the time), 5 (most of the time), and 6 (all of the time). The raw item score was summed by dimension, and the resulting number was converted to a reverse scale of 0 to 100 using the formula 100*(maximum dimension score – score)/5* items in dimension. A higher score correlated with a better quality of life. The MSQ domain scores were reported as change from baseline.

The EuroQol 5-Dimension Questionnaire (EQ-5D) measures the respondent’s health state on 5 dimensions: mobility, self-care, usual activities, pain/discomfort, and anxiety/depression [33]. Each dimension is assessed on a 3-point scale; level 1 (no problems), level 2 (some problems), and level 3 (extreme problems). The current health state is determined using a visual analog scale that ranges from 0 (worst imaginable health state) to 100 (best imaginable health state). The EQ-5D total score is derived from the health state code, which is the combination of levels from each of the 5 dimensions. The score is a continuous range from − 0.59 to 1.00, with 1.00 signifying full health and 0 signifying death. Negative scores indicate a health state worse than death.

#### Adverse drug reactions

The safety and tolerability of long-term onabotulinumtoxinA treatment was evaluated by documenting adverse drug reactions (ADRs) and serious ADRs in the electronic case report form. An ADR was defined as a noxious and unintended response to any treatment administered at a therapeutic dose; an ADR did not necessarily have to be considered related to medical treatment, but a causal relationship between a medical treatment and the event was at least a reasonable possibility. A serious ADR was defined as an ADR that resulted in death, was life threatening, resulted in hospitalisation or prolongation of hospitalisation, resulted in persistent or significant disability and/or incapacity, or was a congenital anomaly or birth defect. Treating physicians recorded ADR frequency, severity (mild, moderate, severe), and causal relation (definite, probable, possible, not assessable) to onabotulinumtoxinA treatment.

### Statistical analysis

All statistical analyses were conducted with SAS version 9.3 (SAS Institute, Inc., Cary, NC). Analysis of demographic, baseline, effectiveness, safety, and tolerability data was done using the safety analysis set, which included all patients who received ≥1 dose of onabotulinumtoxinA. Analysis of headache days, MSQ, and EQ-5D was performed on all patients who completed the 24-month study period. If any EQ-5D dimension score or question on the MSQ was missing, the affected dimension score, as well as the total score, was described as missing. Descriptive statistics were used for continuous variables; frequencies and percentages were provided for categorical data. Changes from baseline in effectiveness variables were tested at the 2-sided 5% level using a nonparametric Wilcoxon signed rank test. If appropriate, Spearman rank correlation coefficients were calculated for effectiveness variables. Incidence rates, including 2-sided 95% CIs, were calculated based on binomial distribution using Clopper-Pearson for all ADRs.

## Results

### Baseline demographics and characteristics

Out of 641 patients who provided informed consent in 78 centres in 7 European countries, 633 were treated at least once with onabotulinumtoxinA. Of those 633 patients, 22.7% (*n* = 144/633) discontinued treatment. Reasons for treatment discontinuation included lack of efficacy in physician’s and/or patient’s opinion (14.2%, *n* = 90/633), patient thinking it was inconvenient to come for treatment (2.7%, *n* = 17/633), side effect(s) or other health problems related to onabotulinumtoxinA treatment (2.4%, *n* = 15/633), or “other” reasons (5.7%, *n* = 36/633, ie, improvement of symptoms, lost to follow-up, and pregnancy). Of the 144 patients discontinuing onabotulinumtoxinA treatment, most did so on or before follow-up visit 4 (*n* = 121; Table 1) because of lack of efficacy (*n* = 75), inconvenience (*n* = 16), side effects (*n* = 9), or “other” reasons (*n* = 21).

**Table 1:** OnabotulinumtoxinA Utilisation

	(*N* = 633)^a^
Treatment sessions per patient	
Mean (SD)	5.5 (3.0)
Min, max	1, 13
Patient discontinued onabotulinumtoxinA treatment, *n* (%)	
Follow-up visit 1	27 (4.3)
Follow-up visit 2	43 (6.8)
Follow-up visit 3	30 (4.7)
Follow-up visit 4	21 (3.3)
Follow-up visit 5	11 (1.7)
Follow-up visit 6	7 (1.1)
Follow-up visit 7	3 (0.5)
Follow-up visit 9	1 (0.2)
Follow-up visit 12	1 (0.2)
Patients attending number of treatment sessions, *n* (%)	
≥ 1	633 (100.0)
≥ 2	573 (90.5)
≥ 3	485 (76.6)
≥ 4	420 (66.4)
≥ 5	371 (58.6)
Injection details per session per patient	
Mean (SD) total dose, U	155.1 (21.4)
Min, max	13, 208
Mean (SD) muscle areas	6.9 (0.6)
Min, max	3.7, 9.0
Mean (SD) injection sites	31.4 (4.1)
Min, max	13, 63^b^
Deviations^c^	
Dose units, U, *n* (%)	
< 155	232 (36.7)
> 195	33 (5.2)
Injection sites, *n* (%)	
< 31	178 (28.1)
> 39	33 (5.2)
Dosing interval, wk, *n* (%)	
< 11	94 (14.8)
> 13	501 (79.1)
> 13 to ≤16	440 (69.5)
> 16	291 (46.0)

^a^Percentages are based on total number of patients who received ≥1 dosage of onabotulinumtoxinA

^b^At the baseline visit (administration visit 1), a maximum of 63 sites per session per patient were injected; across all other administration visits the maximum injection sites per patient was 47

^c^A deviation was defined as a change from the recommended injection paradigm: 155 dose units, 31 injection sites, dosing interval between 11 and 13 weeks. A patient with a deviation at any time during the observation period was included in the deviation category; more than 1 reason for a deviation from the recommended treatment paradigm was allowed, and patients were included in as many categories as required to describe any deviations over the entire study duration. Categories are not mutually exclusive

Mean (SD) age was 45.4 (11.7) years, 85.3% (*n* = 540/633) were women, and mean (SD) body mass index was 24.7 (4.8) kg/m^2^. Mean (SD) age at headache onset was 18.2 (9.9) years. Mean (SD) time since first diagnosis was 242.9 (154.2) months for migraine and 67.4 (96.1) months for CM (Table 1). Mean (SD) number of monthly headache days at baseline was 20.6 (5.4).

Out of 633 patients, only 10% (*n* = 63/633) had received onabotulinumtoxinA previously as a headache or migraine treatment; the remainder were onabotulinumtoxinA-naive. Most patients had previously received beta blockers (71.6%; *n* = 453/633), antidepressants (70.3%; *n* = 445/633), or antiepileptics (69.7%; *n* = 441/633; Table 2). The use of calcium channel blockers (29.9%, *n* = 189/633) was less common. In the 26 weeks before baseline, 84.4% (*n* = 534/633) of patients had been prescribed medication for acute treatment of headache, and 63.5% (*n* = 402/633) had been prescribed preventive medication. At the baseline visit, the most frequently used acute headache medications were sumatriptan (24.0%; *n* = 152/633) and ibuprofen (19.4%; *n* = 123/633; Table 2). The most frequently used preventive medication was topiramate (16.9%; *n* = 107/633). At baseline, 41.4% (*n* = 262/633) of patients were classified by their physician as overusing their headache medications.

**Table 2:** Baseline demographics and characteristics

	(*N* = 633)^a^
Mean (SD) age, y	45.4 (11.7)
Female, *n* (%)	540 (85.3)
Mean (SD) BMI, kg/m^2^	24.7 (4.8)
Headache diagnosis history, *n* (%)^b^	
CM	580 (91.6)
Migraine	453 (71.6)
Medication overuse^c^	229 (36.2)
Tension headache	155 (24.5)
Chronic daily headache	82 (13.0)
Chronic tension-type headache	76 (12.0)
Menstrual headache or menstrual migraine	63 (10.0)
Stress headache	57 (9.0)
Intractable/refractory migraine of headache	46 (7.3)
Cluster headache	15 (2.4)
Sinus headache	7 (1.1)
Hemicrania continua	1 (0.2)
Newly daily persistent headache	1 (0.2)
Other	16 (2.5)
Mean (SD) age of onset of headache, y^d^	18.2 (9.9)
Mean (SD) time since first diagnosis of migraine, mo	242.9 (154.2)
Mean (SD) time since first diagnosis of CM, mo	67.4 (96.1)
Previous headache medications, *n* (%)^b^	
Beta blockers	453 (71.6)
Antidepressants	445 (70.3)
Antiepileptics	441 (69.7)
Calcium channel blockers	189 (29.9)
Botulinum toxin	63 (10.0)
Most frequently prescribed acute headache medications in the last 26 weeks before baseline and being used at baseline, *n* (%)	
Sumatriptan	152 (24.0)
Ibuprofen	123 (19.4)
Zolmitriptan	79 (12.5)
Rizatriptan	77 (12.2)
Most frequently prescribed preventive headache medication in 26 weeks before baseline and being used at baseline, *n* (%)	
Topiramate	107 (16.9)

*BMI* = body mass index, *CM* = chronic migraine

^a^Percentages are based on total number of patients who received ≥1 dosage of onabotulinumtoxinA

^b^Multiple answers were possible; diagnoses were those reported by the study investigator in the period leading up to study enrolment, patients with migraine or chronic daily headache were reclassified as CM, if appropriate, before enrolment

^c^Includes any diagnosis of medication overuse, rebound, or analgesic overuse headache

^d^Based on patient recollection

### OnabotulinumtoxinA utilisation

Study treatment and onabotulinumtoxinA utilisation details are presented in Table 1. There were a total of 3499 treatment sessions and a mean (SD) of 5.5 (3.0) treatment sessions per patient. Out of 633 patients, 100.0% (*n* = 633/633) had ≥1 treatment session, 90.5% (*n* = 573/633) had ≥2, 76.6% (*n* = 485/633) had ≥3, 66.4% (*n* = 420/633) had ≥4, and 58.6% (*n* = 371/633) had ≥5 treatment sessions. The median time from baseline to administration visit 8 was 21.7 months; however, some patients (*n* = 7 [1.1%]) had as many as 13 treatment sessions during the study duration. For each patient, there was a mean (SD) total dose of 155.1 (21.4) U injected per session, which was distributed over a mean (SD) of 31.4 (4.1) injection sites among a mean (SD) number of 6.9 (0.6) muscle areas (Table 1). Most patients received injections into the recommended muscle areas, which included bilateral injections into 6 muscle areas (frontalis, corrugator, occipitalis, temporalis, trapezius, cervical; ≥92%) and one midline injection (procerus; ≥91.0%) from administration visits 1 through 8 (Table 3).

**Table 3:** Muscle Areas Injected With OnabotulinumtoxinA Stratified by Administration Session

Muscle Areas Injected, n (%)^a^	Admin 1 *N* = 633	Admin 2 N = 573	Admin 3 N = 485	Admin 4 N = 420	Admin 5 N = 371	Admin 6 *N* = 329	Admin 7 *N* = 275	Admin 8 *N* = 200
Right side								
Frontalis	630 (99.5)	570 (99.5)	481 (99.2)	417 (99.3)	369 (99.5)	326 (99.1)	274 (99.6)	199 (99.5)
Corrugator	623 (98.4)	560 (97.7)	472 (97.3)	404 (96.2)	361 (97.3)	321 (97.6)	268 (97.5)	191 (95.5)
Occipitalis	625 (98.7)	565 (98.6)	475 (97.9)	410 (97.6)	361 (97.3)	323 (98.2)	268 (97.5)	193 (96.5)
Temporalis	631 (99.7)	570 (99.5)	482 (99.4)	416 (99.0)	368 (99.2)	327 (99.4)	273 (99.3)	198 (99.0)
Trapezius	619 (97.8)	562 (98.1)	477 (98.4)	412 (98.1)	366 (98.7)	324 (98.5)	273 (99.3)	192 (96.0)
Cervical	609 (96.2)	546 (95.3)	465 (95.9)	406 (96.7)	357 (96.2)	318 (96.7)	259 (94.2)	184 (92.0)
Other	67 (10.6)	53 (9.2)	44 (9.1)	42 (10.0)	35 (9.4)	29 (8.8)	29 (10.5)	23 (11.5)
Left side								
Frontalis	628 (99.2)	570 (99.5)	480 (99.0)	418 (99.5)	370 (99.7)	327 (99.4)	274 (99.6)	199 (99.5)
Corrugator	621 (98.1)	559 (97.6)	471 (97.1)	405 (96.4)	361 (97.3)	320 (97.3)	267 (97.1)	191 (95.5)
Occipitalis	622 (98.3)	565 (98.6)	474 (97.7)	411 (97.9)	361 (97.3)	323 (98.2)	267 (97.1)	193 (96.5)
Temporalis	629 (99.4)	570 (99.5)	481 (99.2)	417 (99.3)	369 (99.5)	328 (99.7)	273 (99.3)	196 (98.0)
Trapezius	618 (97.6)	563 (98.3)	476 (98.1)	412 (98.1)	366 (98.7)	325 (98.8)	273 (99.3)	191 (95.5)
Cervical	608 (96.1)	546 (95.3)	465 (95.9)	406 (96.7)	357 (96.2)	317 (96.4)	258 (93.8)	184 (92.0)
Other	66 (10.4)	52 (9.1)	44 (9.1)	40 (9.5)	36 (9.7)	29 (8.8)	29 (10.5)	23 (11.5)
Midline								
Frontalis	0	0	1 (0.2)	0	0	0	0	0
Procerus	584 (92.3)	534 (93.2)	454 (93.6)	394 (93.8)	352 (94.9)	310 (94.2)	256 (93.1)	182 (91.0)
Other	0	0	0	0	0	0	0	0

^a^Percentages are based on the number of patients who received onabotulinumtoxinA at that administration session

In total, 228 (36.0%) patients received additional injections according to the follow-the-pain strategy during a total of 738 sessions. Those patients received an additional mean (SD) dose of 26.4 (13.8) U per session, distributed over a mean (SD) number of 1.9 (0.9) muscle areas and 5.3 (3.0) injection sites.

A deviation from treatment adherence was defined as a change from the recommended injection paradigm of 155 to 195 U, 31 to 39 injection sites, with a dosing interval between 11 and 13 weeks. The majority of deviations were in the dosing interval (ie, < 11 weeks or > 13 weeks), with 440 patients (69.5%) receiving ≥1 treatment after 13 weeks but before 16 weeks and an additional 291 patients (46.0%) receiving ≥1 treatment after 16 weeks (Table 1).

### Outcome measures

#### Use of acute and other preventive treatment

Between baseline and follow-up visit 1, 119 acute treatment changes were made for 71 patients (11.7%), primarily dose reductions or the start of a new acute treatment. During the study, the number and percentage of patients with changes in their acute treatment decreased (Table 4). Similarly, between baseline and follow-up visit 1, 70 preventive treatment changes were made for 54 patients (8.9%), primarily the start of a new preventive treatment or the discontinuation of a preventive treatment. For the first 5 follow-up visits, the percentage of patients having a change in preventive treatment remained relatively stable and then decreased through to the end of the study.

**Table 4:** Change in Acute and Preventive Treatments Between Visits^a^

	Preventive Treatment			Acute Treatment		
	FU 1	FU 5	FU Last	FU 1	FU 5	FU Last
Patients with any changes, *n* (%)^b^	54 (8.9)	30 (7.9)	42 (6.9)	71 (11.7)	12 (3.2)	22 (3.6)
Number of treatments changed, *n*	70	42	58	119	19	36
Started, *n* (%)^c^	30 (42.9)	27 (64.3)	32 (55.2)	50 (42.0)	5 (26.3)	18 (50.0)
Discontinued, *n* (%)^c^	25 (35.7)	10 (23.8)	16 (27.6)	21 (17.6)	2 (10.5)	14 (38.9)
Dose decreased, *n* (%)^c^	11 (15.7)	2 (4.8)	6 (10.3)	64 (53.8)	5 (26.3)	8 (22.2)
Dose increased, *n* (%)^c^	12 (17.1)	6 (14.3)	9 (15.5)	5 (4.2)	7 (36.8)	4 (11.1)

*FU* = follow-up visit; any visit after baseline regardless of whether onabotulinumtoxinA was administered

^a^Change reflects the change between the visit indicated and the visit immediately prior

^b^Percentage is based on the number of patients presenting at the follow-up visit

^c^Percentage is based on the number of medications that were changed since the previous visit. A given treatment change may be associated with 1 or more subcategory (started, discontinued, dose decreased, dose increased)

### Headache-day frequency

**Fig. 1:**
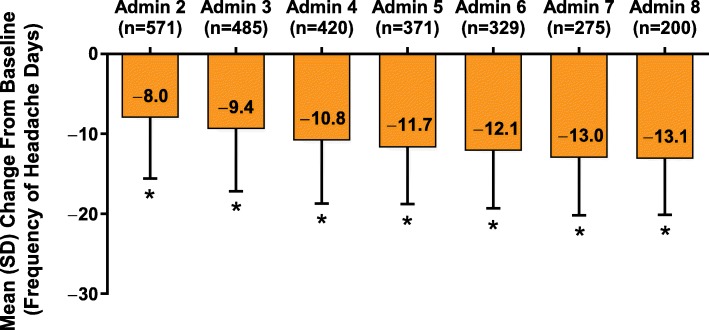
Mean change from baseline in frequency of headache days. Patient-reported estimate of number of days in a month with a headache (≥4 h) at each administration visit through visit 8.^†^ Mean (SD) headache-day frequency at baseline was 20.6 (5.4) days per month. *P < 0.001 Wilcoxon signed rank test for change versus baseline (level of significance: 5%). ^†^The numbers of patients in administration (Admin) visits 9–13 were as follows: Admin 9, *n* = 119; Admin 10, *n* = 50; Admin 11, *n* = 24; Admin 12, *n* = 13; Admin 13, *n* = 7. Mean changes from baseline for Admin visits 9–13 were each significant (Admin 9–12, *P* < 0.001; Admin 13, *P* = 0.016)

At baseline, the mean (SD) headache-day frequency was 20.6 (5.4), and at administration visit 8 it was 7.4 (6.6). Statistically significant reductions (*P* < 0.01) from baseline were observed in headache-day frequency at all postbaseline visits through administration visit 8 (Fig. 1). Although the mean changes from baseline for each administration visit 9 to 13 were significant (admin 9–12, *P* < 0.001; admin 13, *P* = 0.016), the numbers of patients in those sessions were small (admin 9, *n* = 119; admin 10, *n* = 50; admin 11, *n* = 24; admin 12, *n* = 13; admin 13, *n* = 7).

#### Migraine-specific quality-of-life questionnaire

**Fig. 2:**
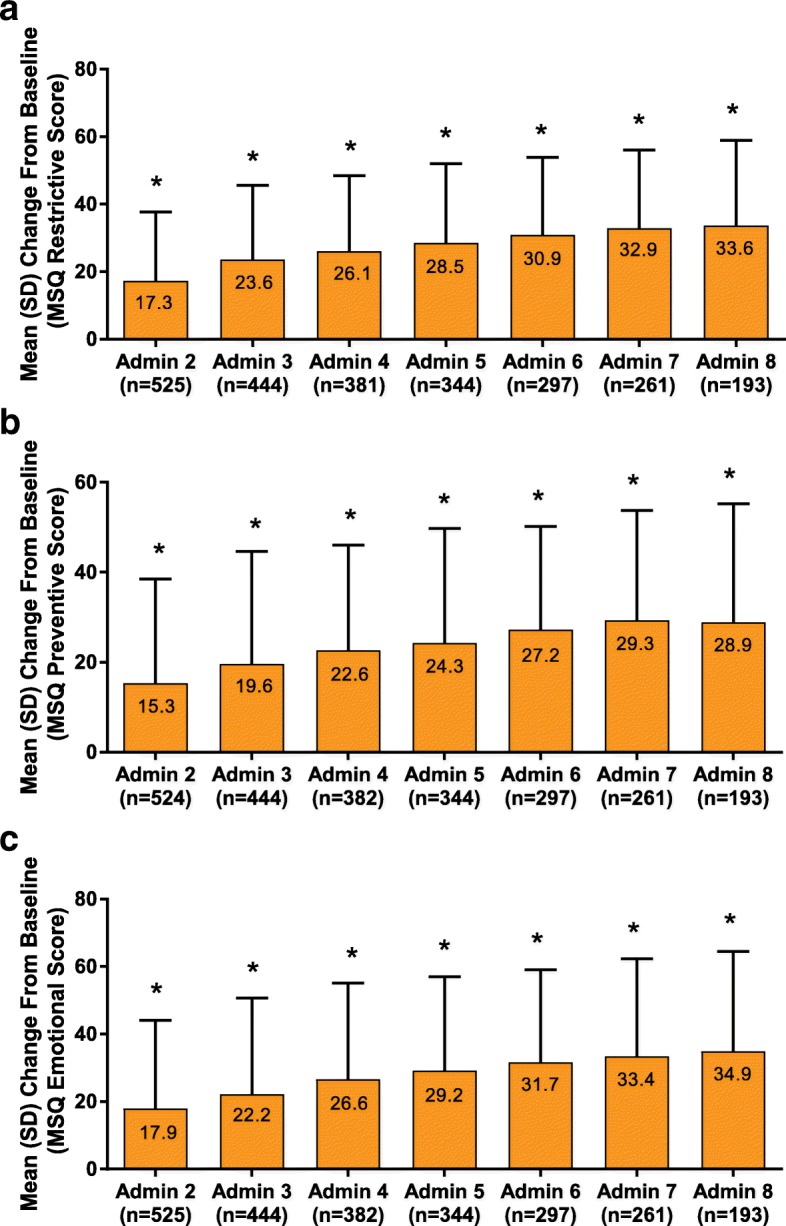
Mean (SD) change from baseline in all MSQ dimensions. **a**) Mean role-function restrictive score at baseline was 36.2 (17.8); **b**) mean role-function preventive score at baseline was 50.2 (22.8); **c)** mean role-function emotional score at baseline was 42.4 (25.6; *P* < 0.001). All dimensions evaluated at administration visit 2 through administration visit 8.^†^ MSQ = Migraine-Specific Quality-of-Life Questionnaire. *P < 0.001 Wilcoxon signed rank test for change versus baseline (level of significance, 5%). ^†^The numbers of patients in administration (Admin) visits 9–13 in all dimensions were as follows: Admin 9, *n* = 117; Admin 10, *n* = 50; Admin 11, *n* = 23; Admin 12, *n* = 13; Admin 13, *n* = 6. Mean changes from baseline for the restrictive and preventive scores Admin visits 9–13 were each significant (Admin 9–12, *P* < 0.001; Admin 13, *P* = 0.031). Mean changes from baseline for the emotional score Admin visits 9–13 were each significant (Admin 9–11, *P* < 0.001; Admin 12, *P* = 0.010; Admin 13, *P* = 0.031)

For the patients who completed questionnaires, statistically significant (*P* < 0.001) changes from baseline were observed in all 3 MSQ domains at all administration visits through administration visit 8 and as early as administration visit 2 (Fig. 2). For the role-function restrictive score, the mean (SD) change from baseline at administration visit 8 was 33.6 (25.3; *P* < 0.001). The role-function preventive score’s mean (SD) change from baseline at administration visit 8 was 28.9 (26.3; *P* < 0.001). The mean (SD) change from baseline for the emotional function score at administration visit 8 was 34.9 (29.6; *P* < 0.001).

#### EuroQol 5-dimension questionnaire

**Fig. 3:**
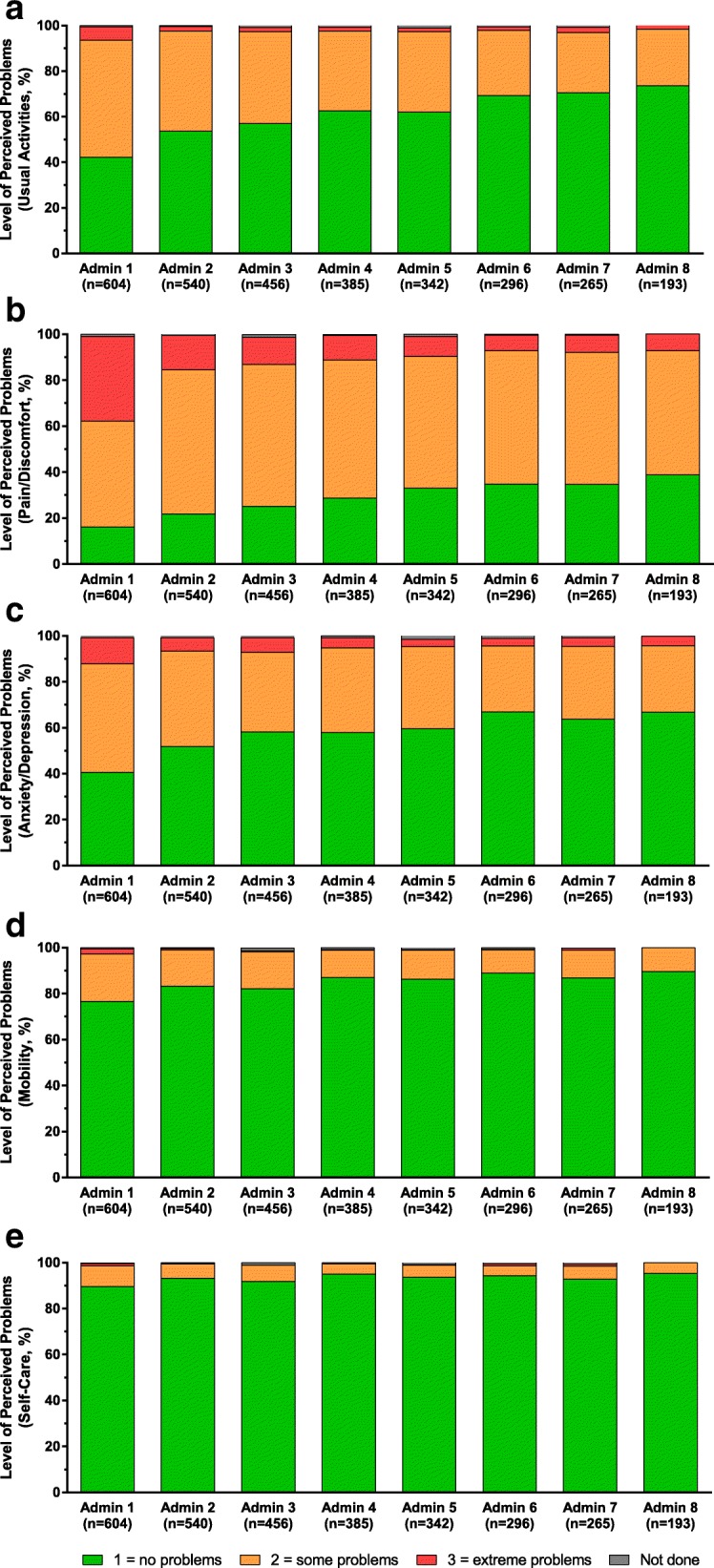
Change from baseline in proportion of patients per level of perceived problems in EQ-5D dimensions. **a**) Usual activities; **b**) pain/discomfort; **c**) anxiety/depression; **d**) mobility; and **e**) self-care. All dimensions evaluated at administration visit 1 through visit 8.* EQ-5D = EuroQol 5-Dimension Questionnaire. *The numbers of patients in administration (Admin) visits 9–13 in all dimensions were as follows: Admin 9, *n* = 119; Admin 10, *n* = 50; Admin 11, *n* = 24; Admin 12, *n* = 13; Admin 13, *n* = 7

**Fig. 4:**
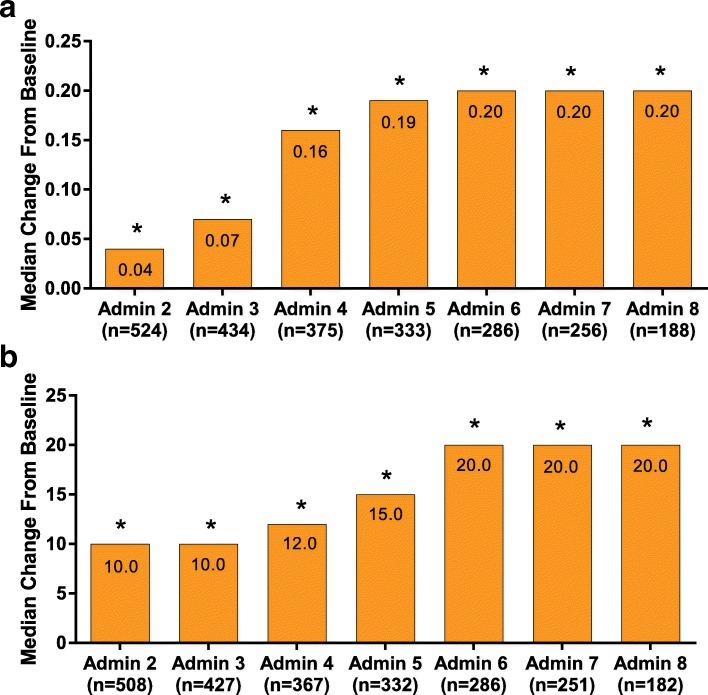
Median change from baseline in EQ-5D total and health state score. **a**) Median EQ-5D total score at baseline was 0.69; **b**) Median EQ-5D health state score at baseline was 50.0. Both scores were evaluated at administration visit 2 through administration visit 8.^†^ EQ-5D = EuroQol 5-Dimension Questionnaire. *P < 0.001 Wilcoxon signed rank test for change versus baseline (level of significance, 5%). ^†^The numbers of patients in administration (Admin) visits 9–13 were as follows: Admin 9, *n* = 111; Admin 10, *n* = 47; Admin 11, *n* = 23; Admin 12, *n* = 12; Admin 13, *n* = 6. Median changes from baseline for Admin visits 9–13 were each significant for the total score (Admin 9–11, *P* < 0.001; Admin 12, *P* = 0.006; Admin 13, *P* = 0.031) and the health state score (Admin 9–11, *P* < 0.001; Admin 12, *P* = 0.002; Admin 13, *P* = 0.031)

Based on the proportions of patients per level of perceived problems, there was a trend toward an improvement in all EQ-5D dimensions between baseline and administration visit 8 for those patients who completed the EQ-5D (Fig. 3). This was particularly evident in the “extreme problems” category in the following domains: usual activities (5.8% at baseline to 1.6% at admin 8), pain/discomfort (36.8% at baseline to 7.3% at admin 8), and anxiety/depression (from 11.4% at baseline to 4.1% at admin 8; Fig. 3). Statistically significant (*P* < 0.001) improvement was observed in the EQ-5D total score as early as administration visits 2 through 8 (Fig. 4a). At administration visit 8, the median EQ-5D total score change from baseline was 0.20 (*P* < 0.001), from a median baseline score of 0.69. Similarly, statistically significant (*P* < 0.001) changes from baseline were observed in health state score at administration visit 2 through administration visit 8 (Fig. 4b). At administration visit 8, the health state score median change from baseline was 20.0 (*P* < 0.001), from a median baseline score of 50.0.

### Safety

Out of 633 patients, 116 (18.3%) reported 267 ADRs (Table 5). Most ADRs were mild (7.1%, *n* = 45/633) to moderate (7.4%, *n* = 47/633) in intensity, with only 24 patients (3.8%) with an ADR of severe intensity. Only 8 patients (1.3%) reported ≥1 serious ADR (events included depression, mental disorder, psychosomatic disease, headache, migraine, vomiting, spinal disorder, spontaneous abortion, and asthma), which typically occurred in patients receiving concomitant headache medication at baseline and throughout the study, making attribution to an individual treatment difficult. No deaths were reported. ADRs in > 2% of patients were eyelid ptosis (5.4%, *n* = 34/633), neck pain (2.8%, *n* = 18/633), and musculoskeletal stiffness (2.7%, *n* = 17/633). Treatment with onabotulinumtoxinA was discontinued in 10 patients (1.6%) because of an ADR, typically as a result of nonserious ADRs such as injection site pain, neck pain, migraine, headache, dizziness, eyelid ptosis, dysphagia, and musculoskeletal pain, weakness, or stiffness. One patient discontinued onabotulinumtoxinA because of a serious ADR (spinal disorder) not considered to be related to treatment. ADRs as evaluated by the treating physician were definite (9.6%, *n* = 61/633), probable (4.9%, *n* = 31/633), possible (2.7%, *n* = 17/633), and not assessable/not assessed (1.6%, *n* = 10/633).

**Table 5:** Summary of adverse drug reactions

	Patients (*N* = 633)^a^
Patients with ADRs, *n* (%)	116 (18.3)
ADRs in > 2% of patients, *n* (%)	
Eyelid ptosis	34 (5.4)
Neck pain	18 (2.8)
Musculoskeletal stiffness	17 (2.7)
ADR intensity, *n* (%)^b^	
Mild	45 (7.3)
Moderate	47 (7.4)
Severe	24 (3.8)
ADR relationship, *n* (%)^b,c^	
Possible	17 (2.7)
Probable	31 (4.9)
Definite	61 (9.6)
Serious ADRs, *n* (%)	8 (1.3)

ADR = adverse drug reaction

^a^Percentages are based on total number of patients

^b^Patients who experienced more than 1 ADR were counted at the maximum intensity or highest causal relationship

^c^Causal relationship as assessed by the investigator

## Discussion

The REPOSE Study aimed to provide real-world observational data regarding the effectiveness, safety, tolerability, and utilisation of onabotulinumtoxinA for the preventive treatment of CM over a 2-year period. Our results demonstrated that long-term, real-life preventive use of onabotulinumtoxinA is effective and well tolerated, with sustained reductions in headache-day frequency and significant improvement in quality of life. No new safety signals were identified with longer-term use and when used with real-world prescribing patterns. Moreover, onabotulinumtoxinA was largely utilised in routine clinical practice as recommended in the SPC and following the injection paradigm established in the PREEMPT Study. The REPOSE Study real-life observations complement the findings of the double-blind, randomised, placebo-controlled PREEMPT Study. The PREEMPT Study reported a significant reduction in headache-day frequency (*P* < 0.001) and significant improvement in all dimensions of the MSQ (*P* < 0.001) compared with baseline [22]. The REPOSE Study was an observational study without strict exclusion criteria; however, patients’ baseline demographics were representative of the CM population seen in routine clinical practice and were comparable to the baseline demographics in the double-blind, randomised, placebo-controlled PREEMPT Study [20–22, 34]. REPOSE Study (vs PREEMPT) patients were primarily women (85.3% vs 87.6%) of similar mean age (45.4 vs 41.1 years old), with a chronic or transformed migraine diagnosis history (91.6%) and baseline headache-day frequency (20.6 vs 19.9) [22].

Akin to this REPOSE Study, other clinical studies have collected data on onabotulinumtoxinA use in a routine clinical setting. A retrospective study in Italy aimed to determine whether onabotulinumtoxinA remained effective after 6 quarterly cycles of treatment in adult CM patients with or without medication overuse, utilising the PREEMPT injection protocol [25]. A total of 47 patients completed all treatment cycles; after the sixth cycle, they reported significant reductions in mean (SD) monthly headache days compared with baseline [25.9 (5.3) vs 6.3 (5.7)] [25]. Similar results were reported by several European prospective studies that observed significant reductions in monthly headache days, migraine days, and improvements in health-related quality-of-life measures [30, 34–36]. In Germany, 96.3% of patients reported benefit after 4 treatment cycles, including reductions in monthly headache days (− 53.7%), reductions in monthly migraine days (− 55.1%), and 1.4 to 2.0 standard deviations improvement in MSQ domain scores [35]. Patients in these single-site, real-life setting studies were similar in baseline demographics to the REPOSE Study and to previous epidemiologic and interventional studies [34, 35]. However, the REPOSE Study is the largest and most diverse observational study reported to date.

An additional aim of the REPOSE Study was to observe routine clinical utilisation of onabotulinumtoxinA. Although treating physicians were trained on the PREEMPT injection paradigm and the onabotulinumtoxinA SPC, the participating physicians in the REPOSE Study were not required to comply. At every treatment session, physicians recorded injection details such as total dose per session and muscle area, total number and location of injection sites, and total muscle areas treated, as well as deviations from the licensed recommendations. Overall, the mean (SD) total dose per treatment session [155.1 (21.4)] and mean (SD) total number of injection sites per session [31.4 (4.1)] were similar to licensed recommendations and consistent with most other real-life onabotulinumtoxinA observational studies [19, 25, 35, 36].

The most frequent deviation reported was in the treatment interval, with a majority of patients (79.1%, *n* = 501) receiving treatment at a dosing interval > 13 weeks and almost half of all patients (46.0%, *n* = 291) receiving treatment at a dosing interval > 16 weeks at least once. We did not capture reasons for deviations in treatment interval because asking this question may have inadvertently influenced the approach to treatment and resulted in more physicians following the recommended treatment protocol. However, we hypothesise that this may be partly attributed to difficulties in scheduling repeat appointments. Despite the increase in dosing interval, outcome measures remained significant, suggesting that increased treatment intervals may also be partly due to a longer duration of effect of onabotulinumtoxinA observed in some patients. Similar results were reported in a European multicentre observational study that aimed to record real-life onabotulinumtoxinA utilisation patterns over a 52-week period [37]. A majority of physicians followed the licensed recommendations regarding muscle areas injected, number of sites (*n* = 31), and total dose per treatment (155 U); 72.8% of patients received treatment at > 13 weeks [37]. Patients reported a high level of satisfaction at the final follow-up interview [37].

Less commonly, patients received onabotulinumtoxinA at dosing intervals < 11 weeks (14.8%; *n* = 94). For a small number of patients (1.1%, *n* = 7), onabotulinumtoxinA was administered 13 times within the 24-month observation period, indicating an average dosing interval of < 8 weeks. The rationale for the < 8-week dosing interval is unknown, but all 7 cases were from study centres in Germany.

During the 24-month observation period in this study, there were no new safety concerns reported, and the incidence and nature of ADRs were comparable to the PREEMPT study [22]. Most ADRs were mild (7.3%) to moderate (7.4%), with the most common being eyelid ptosis (5.4%), neck pain (2.8%), and musculoskeletal stiffness (2.7%). Only 22.7% of patients chose to discontinue onabotulinumtoxinA treatment. The most frequent reason for discontinuation was lack of efficacy in the physician’s and/or patient’s opinion (14.2%).

A real-life observational study provides outcomes that promote an increased understanding of use of the treatment in clinical practice. For example, in the REPOSE Study, treating physicians were trained in the PREEMPT injection paradigm but were not required to comply. Nonetheless, results indicate that overall injection patterns in routine clinical care were similar to licensed recommendations. Similarly, there were variations in dosing intervals that may have been attributable to difficulty in scheduling treatment appointments within the recommended 12-week interval or to a duration of effect of onabotulinumtoxinA longer than 12 weeks in some patients. However, outcome measures remained consistent despite deviations in dosing interval. In addition, other than recent treatment with or contraindications to onabotulinumtoxinA, the REPOSE Study had no strict exclusion criteria, which should have led to a patient population that was representative of the CM general population; indeed, demographics collected at baseline were similar to epidemiologic findings.

Nonetheless, there are also limitations inherent to an observational study in a real-life clinical setting. Observational studies typically have less monitoring and are more reliant on the healthcare professional accurately entering study-related data. This could potentially result in more data discrepancies than in a clinical trial. However, outcome measures and safety and tolerability were similar to those seen in the PREEMPT clinical trials, suggesting that the data recording was robust in the REPOSE Study. Discontinuation of onabotulinumtoxinA treatment during the study due to lack of efficacy may have resulted in an enriched patient population that could potentially skew the outcome measures. In addition, these results represent real-world treatment conditions where many patients were likely taking concomitant preventive medications, which should be taken into account when interpreting these data. Furthermore, study outcomes such as headache-day frequency, MSQ, and EQ-5D were self-reported and therefore dependent on the memory and perception of the patient. Poor recollection could introduce improper data or lead to missing data. Nevertheless, outcome data were similar to previous clinical and real-life studies [25, 34, 35, 38]. Lastly, whereas the EQ-5D is not a migraine-specific health state measure, it has been used to evaluate a number of chronic disease states associated with disabling pain, including rheumatoid arthritis, osteoarthritis of the knee, back pain, and CM [33, 38]. We could have used the unidimensional visual analog scale to measure pain intensity [39]; however, the use of this tool is not common in migraine studies [40]. We used the EQ-5D because it provided a broader assessment of health status [33].

## Conclusions

The results of this open-label, prospective, noninterventional study demonstrate that long-term (2-year) routine clinical use of onabotulinumtoxinA as a preventive medication for CM is efficacious and safe, with sustained reductions in headache-day frequency and significant improvement in quality of life. Moreover, and with the exception of dose interval, onabotulinumtoxinA was utilised in routine clinical practice as recommended in the SPC and following the injection paradigm established in the PREEMPT Study. The safety profile remained favourable, and no new safety concerns were observed with long-term use.

## Abbreviations

ADR: adverse drug reaction
CM: chronic migraine
COMPEL: Chronic Migraine OnabotulinuMtoxinA Prolonged Efficacy open Label
EM: episodic migraine
EQ-5D: EuroQol 5-Dimension Questionnaire
MSQ: Migraine-Specific Quality-of-Life Questionnaire
PASS: Post-Authorization Safety Study
PREEMPT: Phase III Research Evaluating Migraine Prophylaxis Therapy
SPC: summary of product characteristics

## References

[1] Schwedt TJ (2014). Chronic migraine. BMJ (Clinical research ed).

[2] Headache Classification Committee of the International Headache Society (2018). The international classification of headache disorders, 3rd edition. Cephalalgia.

[3] Natoli JL, Manack A, Dean B, Butler Q, Turkel CC, Stovner L (2010). Global prevalence of chronic migraine: a systematic review. Cephalalgia.

[4] Stewart WF, Wood GC, Manack A, Varon SF, Buse DC, Lipton RB (2010). Employment and work impact of chronic migraine and episodic migraine. J Occup Environ Med.

[5] Lanteri-Minet M, Duru G, Mudge M, Cottrell S (2011). Quality of life impairment, disability and economic burden associated with chronic daily headache, focusing on chronic migraine with or without medication overuse: a systematic review. Cephalalgia.

[6] Blumenfeld AM, Varon SF, Wilcox TK, Buse DC, Kawata AK, Manack A (2011). Disability, HRQoL and resource use among chronic and episodic migraineurs: results from the international burden of migraine study (IBMS). Cephalalgia.

[7] Buse DC, Scher AI, Dodick DW, Reed ML, Fanning KM, Manack Adams A (2016). Impact of migraine on the family: perspectives of people with migraine and their spouse/domestic partner in the CaMEO study. Mayo Clin Proc.

[8] Bigal ME, Serrano D, Reed M, Lipton RB (2008). Chronic migraine in the population: burden, diagnosis, and satisfaction with treatment. Neurology.

[9] Bloudek LM, Stokes M, Buse DC, Wilcox TK, Lipton RB, Goadsby PJ (2012). Cost of healthcare for patients with migraine in five European countries: results from the international burden of migraine study (IBMS). J Headache Pain.

[10] Dodick DW, Loder EW, Manack Adams A, Buse DC, Fanning KM, Reed ML (2016). Assessing barriers to chronic migraine consultation, diagnosis, and treatment: results from the chronic migraine epidemiology and outcomes (CaMEO) study. Headache.

[11] World Health Organization. Headache disorders fact sheet (2016) [Available from: http://www.who.int/mediacentre/factsheets/fs277/en/]

[12] Starling AJ, Dodick DW (2015). Best practices for patients with chronic migraine: burden, diagnosis, and management in primary care. Mayo Clin Proc.

[13] Blumenfeld AM, Aurora SK, Laranjo K, Papapetropoulos S (2015). Unmet clinical needs in chronic migraine: rationale for study and design of COMPEL, an open-label, multicenter study of the long-term efficacy, safety, and tolerability of onabotulinumtoxinA for headache prophylaxis in adults with chronic migraine. BMC Neurol.

[14] Stovner LJ, Linde M, Gravdahl GB, Tronvik E, Aamodt AH, Sand T (2014). A comparative study of candesartan versus propranolol for migraine prophylaxis: a randomised, triple-blind, placebo-controlled, double cross-over study. Cephalalgia.

[15] Davies B, Gaul C, Martelletti P, Garcia-Monco JC, Brown S (2017). Real-life use of onabotulinumtoxinA for symptom relief in patients with chronic migraine: REPOSE study methodology and baseline data. J Headache Pain.

[16] Topamax. Buckinghamshire, UK: Janssen-Cilag Limited; 2017

[17] Topamax. Titusville, NJ: Janssen Pharmaceuticals, Inc.; 2017

[18] Diener HC, Bussone G, Van Oene JC, Lahaye M, Schwalen S, Goadsby PJ (2007). Topiramate reduces headache days in chronic migraine: a randomized, double-blind, placebo-controlled study. Cephalalgia.

[19] Botox. Westport, County Mayo, Ireland: Allergan Pharmaceuticals Ireland; 2014

[20] Diener HC, Dodick DW, Aurora SK, Turkel CC, DeGryse RE, Lipton RB (2010). OnabotulinumtoxinA for treatment of chronic migraine: results from the double-blind, randomized, placebo-controlled phase of the PREEMPT 2 trial. Cephalalgia.

[21] Aurora SK, Dodick DW, Turkel CC, DeGryse RE, Silberstein SD, Lipton RB (2010). OnabotulinumtoxinA for treatment of chronic migraine: results from the double-blind, randomized, placebo-controlled phase of the PREEMPT 1 trial. Cephalalgia.

[22] Aurora SK, Winner P, Freeman MC, Spierings EL, Heiring JO, DeGryse RE (2011). OnabotulinumtoxinA for treatment of chronic migraine: pooled analyses of the 56-week PREEMPT clinical program. Headache.

[23] Blumenfeld A, Silberstein SD, Dodick DW, Aurora SK, Turkel CC, Binder WJ (2010). Method of injection of onabotulinumtoxinA for chronic migraine: a safe, well-tolerated, and effective treatment paradigm based on the PREEMPT clinical program. Headache.

[24] Blumenfeld AM, Stark RJ, Freeman MC, Orejudos A, Manack Adams A (2018). Long-term study of the efficacy and safety of onabotulinumtoxinA for the prevention of chronic migraine: COMPEL study. J Headache Pain.

[25] Santoro A, Fontana A, Miscio AM, Zarrelli MM, Copetti M, Leone MA (2017). Quarterly repeat cycles of onabotulinumtoxinA in chronic migraine patients: the benefits of the prolonged treatment on the continuous responders and quality-of-life conversion rate in a real-life setting. Neurol Sci.

[26] Cernuda-Morollon E, Ramon C, Larrosa D, Alvarez R, Riesco N, Pascual J (2015). Long-term experience with onabotulinumtoxinA in the treatment of chronic migraine: what happens after one year?. Cephalalgia.

[27] Guerzoni S, Pellesi L, Baraldi C, Cainazzo MM, Negro A, Martelletti P (2017). Long-term treatment benefits and prolonged efficacy of onabotulinumtoxinA in patients affected by chronic migraine and medication overuse headache over 3 years of therapy. Front Neurol.

[28] Negro A, Curto M, Lionetto L, Crialesi D, Martelletti P (2015). OnabotulinumtoxinA 155 U in medication overuse headache: a two years prospective study. SpringerPlus.

[29] Negro A, Curto M, Lionetto L, Martelletti P (2016). A two years open-label prospective study of onabotulinumtoxinA 195 U in medication overuse headache: a real-world experience. J Headache Pain.

[30] Khalil M, Zafar HW, Quarshie V, Ahmed F (2014). Prospective analysis of the use of onabotulinumtoxinA (Botox) in the treatment of chronic migraine; real-life data in 254 patients from Hull, U.K. J Headache Pain.

[31] Martin BC, Pathak DS, Sharfman MI, Adelman JU, Taylor F, Kwong WJ (2000). Validity and reliability of the migraine-specific quality of life questionnaire (MSQ version 2.1). Headache.

[32] Cole JC, Lin P, Rupnow MF (2007). Validation of the migraine-specific quality of life questionnaire version 2.1 (MSQ v. 2.1) for patients undergoing prophylactic migraine treatment. Qual Life Res.

[33] Walters SJ, Brazier JE (2005). Comparison of the minimally important difference for two health state utility measures: EQ-5D and SF-6D. Qual Life Res.

[34] Pedraza MI, de la Cruz C, Ruiz M, Lopez-Mesonero L, Martinez E, de Lera M (2015). OnabotulinumtoxinA treatment for chronic migraine: experience in 52 patients treated with the PREEMPT paradigm. SpringerPlus.

[35] Kollewe K, Escher CM, Wulff DU, Fathi D, Paracka L, Mohammadi B (2016). Long-term treatment of chronic migraine with onabotulinumtoxinA: efficacy, quality of life and tolerability in a real-life setting. J Neural Transm.

[36] Vikelis M, Argyriou AA, Dermitzakis EV, Spingos KC, Mitsikostas DD (2016) Onabotulinumtoxin-A treatment in Greek patients with chronic migraine. J Headache Pain 17:84 10.1186/s10194-016-0676-z PMC5026980 27640152

[37] Matharu M, Pascual J, Nilsson Remahl I, Straube A, Lum A, Davar G (2017). Utilization and safety of onabotulinumtoxinA for the prophylactic treatment of chronic migraine from an observational study in Europe. Cephalalgia.

[38] Maasumi K, Thompson NR, Kriegler JS, Tepper SJ (2015). Effect of onabotulinumtoxinA injection on depression in chronic migraine. Headache.

[39] McCormack HM, Horne DJ, Sheather S (1988). Clinical applications of visual analogue scales: a critical review. Psychol Med.

[40] Herd CP, Tomlinson CL, Rick C, Scotton WJ, Edwards J, Ives N (2018). Botulinum toxins for the prevention of migraine in adults. Cochrane Database Syst Rev.

